# A chromosome-level genome assembly of East Asia endemic minnow *Zacco platypus*

**DOI:** 10.1038/s41597-024-03163-w

**Published:** 2024-03-27

**Authors:** Xiaojun Xu, Jing Chen, Wenzhi Guan, Baolong Niu, Shaokui Yi, Bao Lou

**Affiliations:** 1 State Key laboratory for Managing Biotic and Chemical Threats to the Quality and Safety of Agro-products, Institute of Hydrobiology, Zhejiang Academy of Agricultural Sciences, Hangzhou, China; 2 Zhejiang Institute of Freshwater Fisheries, Huzhou, China; 3 School of Life Sciences, Huzhou University, Huzhou, China

**Keywords:** Evolution, Genetics

## Abstract

*Zacco platypus* is an endemic colorful freshwater minnow that is intensively distributed in East Asia. In this study, two adult female individuals collected from Haihe River basin were used for karyotypic study and genome sequencing, respectively. The karyotype formula of *Z. platypus* is 2N = 48 = 18 M + 24SM/ST + 6 T. We used PacBio long-read sequencing and Hi-C technology to assemble a chromosome-level genome of *Z. platypus*. As a result, an 814.87 Mb genome was assembled with the PacBio long reads. Subsequently, 98.64% assembled sequences were anchored into 24 chromosomes based on the Hi-C data. The chromosome-level assembly contained 54 scaffolds with a N50 length of 32.32 Mb. Repeat elements accounted for 52.35% in genome, and 24,779 protein-coding genes were predicted, with 92.11% were functionally annotated with the public databases. BUSCO analysis yielded a completeness score of 96.5%. This high-quality genome assembly provides valuable resources for future functional genomic research, comparative genomics, and evolutionary studies of genus *Zacco*.

## Background & Summary

*Zacco platypus* is one of the endemic colorful minnows that are widespread in the freshwater ecosystems of East Asia^1^. It’s often used to assess the contaminant on aquatic environment of North Korea, South Korea and China as a test model and indicator species^2–5^. Recently, for the unique nupitial characteristics (sexual dimorphism and dichromatism, elongated anal fin and nuptial tubercles of the male), *Z. platypus* has become an important emerging native ornamental fish in China.

*Z. platypus* has undergone a long and complex taxonomic history. As the type species of genus *Zacco*, *Z. platypus* was first described from Nagasaki, Japan^6^. It was successively placed in Cyprinidae, Leuciscinae, *Zacco*^7^ and Cyprinidae, Danioninae, *Zacco*^8^. After a series of revisions^9–12^, it is currently assigned into Xenocyprididae, Opsariichthyinae, *Zacco*^13^. The genus *Zacco* was established in 1902, and the discriminating criterion for *Zacco* and *Opsariichthys* was that “*Opsariichthys* is presence of peculiar notched jaws, but it is absent in *Zacco*”^14^. After a series of taxonomic studies^15–19^, the diagnostic features of *Zacco* were modified to: (1) “the nuptial tubercles on the cheeks are united basally to from a plate in male” commented by Jordan and Hubb^20^; and (2) the fused light green lateral crossbars into fewer large patches which can be well separate from other members of *Opsariichthys*^21^. A series of population genetics research using mitochondrial cytochrome b (Cytb) fragments and intron polymorphism revealed that Chinese *Z. platypus* contained multiple molecular lineages^22–24^. The morphological comparisons and genetic analyses using AFLP makers indicated that *O. evolans* was a valid species, which had been proposed as a synonym of *Z. platypus*^25^. Particularly, *O. evolans* and *O. acutipinnis* had been reported as synonym species of *Z. platypus*^8^. Therefore, molecular lineages from upper-middle Yangtze and Pearl River basins should be regarded as the members for *O. acutipinnis*-*O. evolans* complex. Consequently, it was once thought that the genus *Zacco* should be restricted only to the type species, *Z. platypus*, which may be merely distributed from Japan to the north of Zhejiang Province, China^26^.

In the last few years, there have been some new opinions on the taxonomy of *Zacco*. Molecular analysis based on three nuclear genes suggested that *Z. acanthogenys* might be a valid species, while no comprehensive diagnostic feature has been reported, other than the red upper iris^27^. More recently, *Z. sinensis* sp. nov and *Z. tiaoxiensi* sp. nov were described by using morphological and mitochondrial data^28,29^. Due to its wide distribution, *Z. platypus* exhibits great morphological flexibility. Our site survey found that the body size and color pattern varied in different river basins of China, and even in different drainages of the same river basin. Limited nuclear genes or mitochondrial markers represent only a small percentage of the genome or are of maternal origin, which may lead to biases when drawing systematic and taxonomic conclusions^30^. Thus, the taxonomy of *Zacco* is still in debate. To facilitate taxonomic and phylogenetic studies of *Zacco* fishes, genome-wide genetic information is urgently needed. Although Xu *et al*. has reported the whole genome of *Z. platypus*^31^, the chromosome-level genome assembly of this species is still unavailable. Here, we assembled a high-quality chromosome-level genome of East Asia endemic minnow *Z*. *platypus*. This new assembly will greatly improve the systematic and taxonomic study of genus *Zacco*. Furthermore, access to the genomic data set will facilitate the use of *Z. platypus* as an indicator organism for assessing the contaminant on aquatic environment.

## Methods

### Sample collection and genome sequencing

A healthy female *Z. platypus* was collected from Xingtai City, Hebei Province of China (37.0750 °N, 113.9221 °E). High-quality genomic DNA was extracted from muscle tissue for genome libraries construction, and then the library construction and sequencing work were completed at Frasergen Co., Ltd. (Wuhan, China). For short-read sequencing, the Illumina Hiseq X-10 platform (Illumina, San Diego, CA, USA) was used to perform paired-end sequencing with an insert size of 300~350 base pairs (bp). For long-read DNA sequencing, the PacBio sequencing was performed on a PacBio Sequel II platform with continuous long-read (CLR) mode.

To anchor scaffolds onto the chromosome, a chromosome conformation capture (Hi-C) library was prepared using muscle tissue. The Hi-C library was constructed following the standard protocol described previously^32^, and sequenced on an Illumina Hiseq X-10 platform (Illumina, San Diego, CA, USA).

In addition, total RNAs from the tissues of muscle, blood, brain, liver, and spleen were extracted for Iso-Seq using Qiagen RNeasy Mini Kit (Qiagen, Hilden, Germany). The RNA samples from 5 tissues were equally mixed. An Iso-Seq cDNA library was constructed according to the PacBio standard protocol with the BluePippin size selection system (Sage Science, MA, USA) and sequenced on the PacBio sequel II platform.

### Karyotypic analysis

**Fig. 1:**
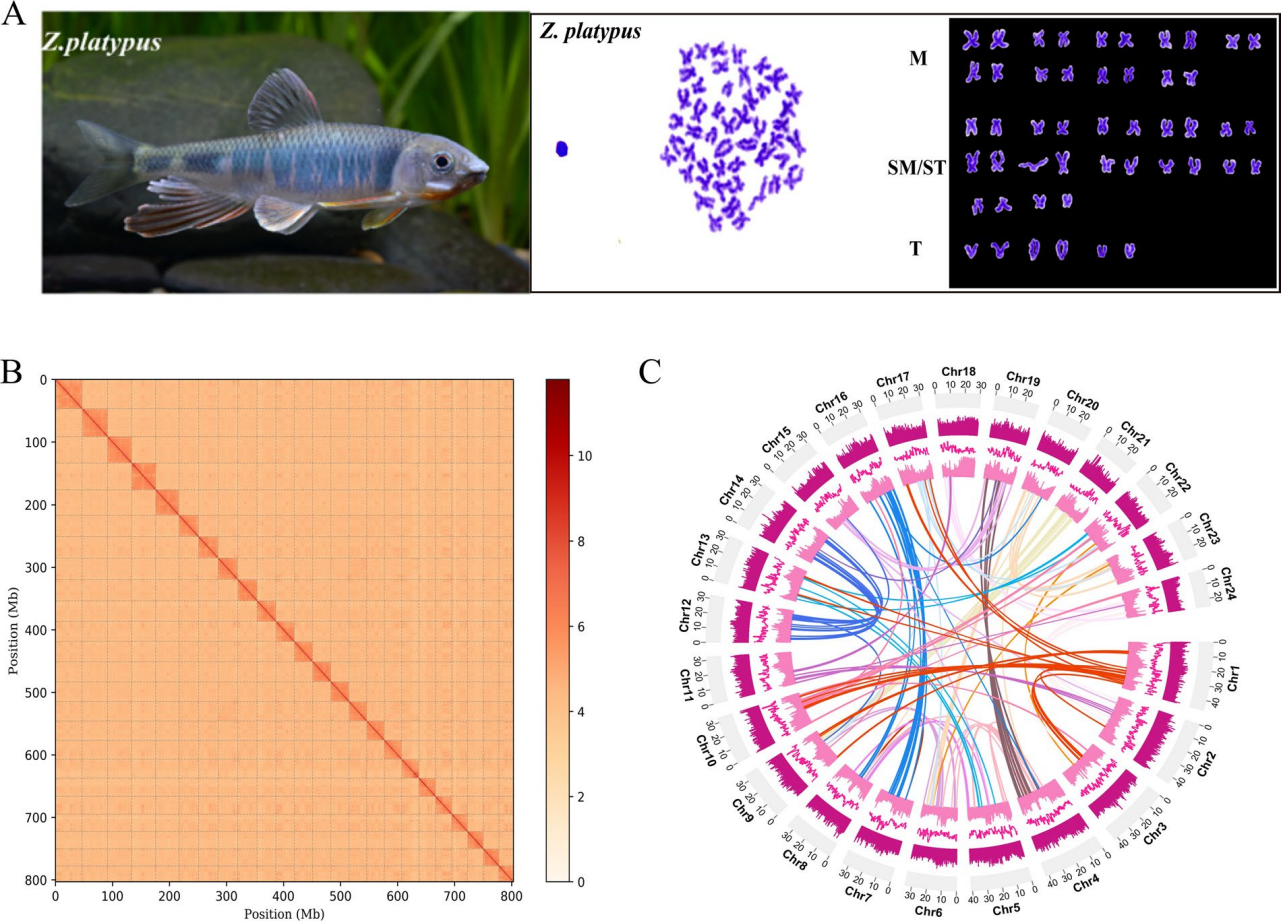
Karyotype and genomic information visualization of *Zacco platypus*. (**A**) The image and karyotype of *Z. platypus*. (**B**) Heat map of interactive intensity between chromosome sequences of *Z. platypus* anchored by Hi-C. (**C**) Circos plot of 24 assembled chromosomes for *Z. platypus* genome. From the outside to the inside, the tracks indicate 24 chromosomes, GC content (bin = 1 Mb), gene density (bin = 1 Mb), repetitive sequence density (bin = 1 Mb) and the major interchromosomal syntenic relationships, respectively.

An adult female *Z. platypus* individual collected from the same location with the sequencing individual was used for karyotyping experiment, according to the published pipeline^33^. Chromosomes were photographed using a Leica DM4 B fluorescence microscope (Leica, Wetzlar, Gemany). Chromosome classifications were made by the standardized nomenclature^34^. The result showed that *Z. platypus* has a chromosome number of 2n = 48 and a karyotype formula of 18 M + 24SM/ST + 6 T (Fig. 1A).

### Genome assembly

The Illumina sequencing produced 84.32 Gb clean data after the quality control (Table 1). The genome size, repeat content and heterozygosity were estimated by *K*-mer analysis with Illumina short reads. Frequencies of *K*-mers (*K = *17) were counted using Jellyfish v2.2.6^35^. The genome size was estimated to be approximately 818.15 Mb, with a heterozygosity of 0.37% and 47.72% of repeat sequences. Then, the genome assembly was conducted with the obtained 172.05 Gb PacBio data using the Falcon assembler v0.3 (Table 1). The draft genome was further polished by gcpp v2.0.2 (https://github.com/PacificBiosciences/gcpp) and pilon v1.22^36^ to improve the quality of genome assembly. This preliminary assembly of *Z. platypus* genome was 814.87 Mb in length with an N50 of 8.10 Mb (Table 2).

**Table 1:** Statistics of sequencing data.

Library types	Platform	Sample	Raw data (Gb)	Clean data (Gb)
WGS short reads	Illumina HiSeq X-10	muscle	107.87	84.32
WGS long reads	Pacbio Sequel II	muscle		172.05
Hi-C	Illumina HiSeq X-10	muscle	157.27	151.17
Iso-Seq	Pacbio Sequel II	Muscle, blood, brain, liver and spleen		77.90

**Table 2:** Statistics of *Zacco platypus* genome assembly.

Assembly and annotation metrics	Number or percentage
Total length (bp)	814,871,113
Contig N50 (bp)	8,100,637
Scaffold N50 (bp)	32,319,397
Longest chromosome (bp)	46,872,128
Shortest chromosome (bp)	25,275,430
GC content (%)	37.82
Hi-C anchored ratio (%)	98.64
Gene number	24,779
Complete BUSCOs ratio (%)	96.30

Subsequently, 151.17 Gb Hi-C data were aligned to the assembly using the Juicer v1.6.2^37^ (Table 1). The contigs were ordered and anchored with Hi-C data using the 3D-DNA^38^ and manually adjusted using Juicebox Assembly Tools v1.11.08^39^. Finally, the Hi-C interaction heatmap demonstrated an excellent quality of the genome assembly (Fig. 1B). Approximately 98.64% of the contig sequences were anchored to 24 chromosomes, which is consistent with the karyotype analysis in this study (Fig. 1A). The Circos^40^ was used to visualize the 24 chromosomes, GC content, gene density, repetitive sequence density and major interchromosomal syntenic relationships (Fig. 1C). The longest and shortest chromosomes were 46.87 Mb and 25.28 Mb in length, respectively (Table 2). The N50 reached 32.32 Mb for the final genome assembly (Table 2). The assembly completeness was evaluated by Benchmarking Universal Single-Copy Orthologs (BUSCO) v3.0.2^41^ with actinopterygii_odb10. We found that 96.30% of BUSCO genes were completely detected in the final assembly.

### Repeat annotation

The repetitive elements in the genome of *Z. platypus* were annotated by using a combination of homology-based and *ab* initio approaches. For the homology-based approach, the repeat sequences were identified with RepeatMasker v4.0.9 and RepeatProteinMasker v4.0.9 (http://www.repeatmasker.org/) using Repbase database (http://www.girinst.org/repbase/). For the *ab* initio approach, RepeatModeler v1.0.11 (http://www.repeatmasker.org/RepeatModeler/) and LTR-FINDER software v1.0.5^42^ were used to build an *ab* initio repeat sequence library, and then RepeatMasker v4.0.9 was used to predict repeat sequences. Furthermore, TRF v4.09^43^ was used to find tandem repeats in the genome. Finally, a total of 426.68 Mb repetitive sequences were identified by combining the *de novo*, and homology-based approaches, accounting for 52.35% of the whole genome (Table 3). In detail, 403.00 Mb (49.45%) of TEs, including 259.97 Mb DNA repeat elements (31.90%), 56.25 Mb long interspersed nuclear elements (LINE, 6.90%), 6.20 Mb short interspersed nuclear elements (SINE, 0.76%), 104.51 Mb long terminal repeat elements (LTR, 12.82%), and 35.45 Mb unknown elements (4.35%) were detected (Table 4).

**Table 3:** Summary of repetitive sequences.

Type	Repeat Size (bp)	% of genome
Tandem Repeat Finder	46,612,949	5.72
Repeat Masker	176,270,521	21.63
Pepeat Protein Mask	41,366,321	5.08
*De novo*	336,905,871	41.34
Total	426,684,759	52.35

**Table 4:** Statistics of repetitive sequence classification results.

Type	Repbase TEs		Protein TEs		Denovo TEs		Combined TEs	
	Length (bp)	% of genome	Length (bp)	% of genome	Length (bp)	% of genome	Length (bp)	% of genome
DNA	116,226,674	14.26	12,773,306	1.57	190,660,672	23.39	259,970,064	31.9
LINE	19,496,315	2.39	13,518,888	1.66	47,140,468	5.78	56,248,936	6.9
SINE	2,651,022	0.33	0	0	3,972,759	0.49	6,203,958	0.76
LTR	32,132,506	3.94	15,085,559	1.85	93,700,362	11.5	104,505,083	12.82
Other	0	0	0	0	0	0	0	0
Unknown	3,139,871	0.39	690	0	32,913,546	4.04	35,447,776	4.35
Total TE	165,986,555	20.37	41,366,321	5.08	327,725,535	40.21	402,995,807	49.45

Note: TEs, transposable elements; LINE, long interspersed nuclear elements; SINE, short interspersed nuclear elements; LTR, long terminal repeats.

### Gene annotation

To obtain high quality protein-coding genes of *Z. platypus* genome, a comprehensive strategy combining homology-based prediction, transcript-based prediction and *de novo* prediction was employed. For the homology-based prediction, protein sequences from *Ancherythroculter nigrocauda* (GCA_036281575.1), *Danio rerio* (GCA_000002035.4), *Onychostoma macrolepis* (GCA_012432095.1), *Carassius auratus* (GCA_003368295.1), *O. bidens* (GWHBEIO00000000) were downloaded from Ensembl database (http://www.ensembl.org) and NGDC database (https://ngdc.cncb.ac.cn/). These sequences were aligned to the *Z. platypus* genome using Exonerate software^44^. Meanwhile, a total of 77.90 Gb clean data was generated with Iso-Seq, and 32,860 transcripts with a mean length of 2582 bp were obtained with the Iso-Seq workflow. For the transcript-based prediction, PASA^45^ was used to annotate gene structure with the full-length trancripts. For the *de novo* prediction, the gene structure was identified with Augustus v3.3^46^ and GenScan v1.0^47^. All data were then integrated using MAKER2^48^. PASA was used to further refine the gene structure based on transcriptome data and a total of 24,779 protein-coding genes were predicted, with average gene length and exon number per gene of 17,588.54 bp and 9.41, respectively (Table 5).

**Table 5:** Statistics of gene prediction.

Gene set	Number	Average gene length (bp)	Average CDS length (bp)	Average exon per gene	Average exon length (bp)	Average intron length (bp)
denovo/AUGUSTUS	20,530	17,896.43	1,643.66	9.6	171.24	1,890.16
denovo/Genscan	25,473	22,412.84	1,628.68	8.81	184.93	2,662.20
homo/A.nigrocauda	56,406	9,546.94	943.63	5.1	185.07	2,098.98
homo/D.rerio	46,647	11,475.05	1,199.98	5.7	210.67	2,188.01
homo/O.macrolepis	48,098	10,706.94	1,098.65	5.73	191.82	2,032.43
homo/C.auratus	49,568	11,165.14	1,168.59	5.58	209.47	2,183.22
homo/O.bidens	54,045	8,417.95	1,001.80	5.01	199.8	1,847.54
trans.orf/Iso-Seq	14,218	19,668.62	1,660.69	10.85	281.1	1,686.59
MAKER	24,980	17,031.24	1,603.61	9.37	240.11	1,764.88
PASA	24,779	17,588.54	1,600.20	9.41	255.49	1,806.64

Gene function annotation was performed by aligning the genes to several databases, including NCBI Nr, Swiss-Prot^49^, Pfam^50^, GO^51^, KEGG^52^, InterPro^53^, and TrEMBL^54^ using BLASTP (e-value ≤ 1e^−5^, max_target_seqs 1). Finally, 22,823 genes accounting for 92.11% of the total were successfully annotated with at least one database (Table 6). The annotated genes contained 91.40% complete and 2.70% fragmented BUSCOs using actinopterygii_odb10, indicating that the annotation has high completeness.

**Table 6:** Statistics of *Zacco platypus* genome annotation.

Database	Number	Percent (%)
InterPro	21088	85.10
GO	16247	65.57
KEGG_ALL	22596	91.19
KEGG_KO	12383	49.97
Swissprot	21115	85.21
TrEMBL	22616	91.27
NR	22799	92.01
Annotated	22823	92.11
Unannotated	1956	7.89
Total	24779	NA

Finally, tRNAscan-SE^55^ and BLASTN was used to predict tRNA and rRNA sequences in the genome, respectively. Additionally, miRNA and snRNA sequences were identified with Infernal program with Rfam^56^. The genomic noncoding RNAs, including 668 microRNAs (miRNAs), 10,272 transfer RNAs (tRNAs), 1332 ribosomal RNAs (rRNAs), and 534 small nuclear RNAs (snRNAs) were identified in the genome (Table 7).

**Table 7:** Statistics of noncoding RNA annotation result.

Type		Copy	Average length (bp)	Total length (bp)	% of genome
miRNA		668	108.2	72,280	0.01
tRNA		10,272	76.09	781,594	0.1
rRNA	rRNA	1,332	151.82	202,223	0.02
	18 S	11	2,432.64	26,759	0
	28 S	5	4,776.80	23,884	0
	5.8 S	0	0	0	0
	5 S	1,316	115.18	151,580	0.02
	8 S	0	0	0	0
snRNA	snRNA	534	136.66	72,977	0.01
	CD-box	113	147.29	16,644	0
	HACA-box	50	157.36	7,868	0
	splicing	364	129.32	47,073	0.01
	scaRNA	7	198.86	1,392	0

## Data Records

All the raw sequencing data utilized in this study were submitted to the National Center for Biotechnology Information (NCBI) SRA (Sequence Read Archive) database under BioProject accession number PRJNA1028840. Specifically, the Illumina WGS data was archived with the accession number SRR26456191^57^, while the PacBio WGS data was deposited with the accession number SRR26456189^58^. The Iso-Seq and Hi-C data sets were archived under the accession numbers SRR26456188^59^ and SRR26456190^60^, respectively. The final chromosome assembly has been deposited at GenBank under the accession number JAYDZZ000000000^61^. The genome annotation file has been deposited at the Figshare^62^.

## Technical Validation

The quality scores across all bases and GC content of the Illumina raw sequencing data were inspected by FastQC v0.11.9 (https://www.bioinformatics.babraham.ac.uk/projects/fastqc/). BUSCO v3.0.2 was used for quantitative assessment of genome assembly and evaluating the completeness of protein-coding annotation with the actinopterygii_odb10^41^.

## Data Availability

All data processing commands and pipelines were carried out in accordance with the instructions and guidelines provided by the relevant bioinformatic software. This study does not involve custom scripts or code.
